# Exploring key components and factors that influence the use of clinical decision- support tools for prescribing to older patients with kidney disease: the perspective of healthcare providers

**DOI:** 10.1186/s12913-024-10568-1

**Published:** 2024-01-23

**Authors:** N Alsalemi, CA Sadowski, K Kilpatrick, N Elftouh, SKD Houle, JP Lafrance

**Affiliations:** 1 Département de pharmacologie et physiologie, Université de Montréal, Montréal, Canada; 2 Centre de recherche de l’Hôpital Maisonneuve-Rosemont, Montréal, Canada; 3 College of Pharmacy, Qatar University, Doha, Qatar; 4 Faculty of Pharmacy and Pharmaceutical Sciences, University of Alberta, Edmonton, Canada; 5 Ingram School of Nursing, McGill University, Montreal, Canada; 6 School of Pharmacy, University of Waterloo, Waterloo, Canada; 7 Service de néphrologie, CIUSSS de l’Est-de-l’Île-de-Montréal, Montréal, Canada

**Keywords:** Clinical decision-support, Cross-sectional survey, Facilitators and barriers, Prescribing, Older adults

## Abstract

**Background:**

Clinical decision-support (CDS) tools are systems that provide healthcare providers (HCPs) with recommendations based on knowledge and patient-specific factors to facilitate informed decisions.

**Objectives:**

To identify the key components of a CDS tool that are most important to HCPs in caring for older adults with kidney disease, and to understand the facilitators and barriers toward using CDS tools in daily clinical practice.

**Methods:**

*Design*: A cross-sectional survey of Canadian HCPs was undertaken. *Data collection*: Participants affiliated with a provincial college, nephrology organization, or advocacy body were contacted. The survey was conducted between August and October 2021. *Instrument*: A 59-item questionnaire was developed and divided into five main domains/themes. Analysis was done descriptively.

**Results:**

Sixty-three participants completed the questionnaire. Physicians (60%) and pharmacists (22%) comprised the majority of the participants. Most of the participants were specialized in nephrology (65%). The most important components in a CDS tool for prescribing to older patients with kidney disease were the safety and efficacy of the medication (89%), the goal of therapy (89%), and patient’s quality of life (87%). 90% were willing to use CDS tools and 57% were already using some CDS tools for prescribing. The majority of the participants selected the validation of CDS tools (95%), accompanying the recommendations by the supporting evidence (84%), and the affiliation of the tools with known organizations (84%), as factors that facilitate the use of CDS tools.

**Conclusion:**

CDS tools are being used and are accepted by HCPs and have value in their assistance in engaging patients in making well-informed decisions.

**Supplementary Information:**

The online version contains supplementary material available at 10.1186/s12913-024-10568-1.

## Introduction

Clinical decision support (CDS) tools are standardized instruments that are intended to support clinical decision making. They are used to achieve best practice by providing tailored recommendations based on clinical protocols, clinician’s knowledge, and patient’s health information [1–3]. Research has linked the use of CDS tools to a number of benefits, including reducing the incidence of prescribing errors, improving adherence to clinical practice guidelines (CPGs), reducing unnecessary lab orders, and workflow improvement [3, 4]. Conversely, CDS tools have also been associated with inadequate documentation, disturbing the communications between the patient and the clinician, and causing unnecessary referrals [3, 4]. 

Older adults usually present with several comorbidities and show heterogeneity and complexity [5]. Applying recommendations from different disease-specific CPGs to each comorbidity of older patients can result in a multitude of conflicting recommendations [6]. This can be challenging for clinicians and could potentially leave the ultimate decision to expert opinion, rather than evidence-based medicine (EBM) [6, 7]. As a result, older patients are at high risk of receiving sub-optimal or inappropriate treatments [7]. Thus, CDS tools are hypothesized to be useful in caring for complex cases with multimorbidity and polypharmacy, including those of older patients. CDS tools can be an effective approach to aid in clinical decision-making and prescribing practices, and have the potential to improve the quality of care and the application of CPGs while providing personalized recommendations [8]. To achieve this goal, CDS tools should be customized for prescribing to older adults. There are several aspects that older adults consider as priorities in their therapy plans [9]. These may include quality of life, goals of therapy, and remaining life expectancy [9]. 

The objectives of this study are to (1) identify the key components of a CDS tool that are most important to HCPs in caring for older adults with kidney disease; and (2) understand the facilitators and barriers toward using CDS tools in daily clinical practice.

## Methods

Study Design: We conducted a cross-sectional survey of HCPs who reside in Canada in the period of August and October 2021.

### Data collection

A 59-item questionnaire was developed and divided into five main domains/themes (Supplementary file). The questionnaire was designed to capture HCPs’ perspectives about the use of CDS tools in their daily clinical practice, covering the following themes: (1) description of the HCPs’ clinical practice, (2) the use of CDS tools in clinical practice (3) key components of CDS tools, (4) barriers and facilitators to the use of CDS tools, and (5) attitudes towards using CDS tools. Items were developed based on literature review, and survey drafts were discussed among the research team members and experts to ensure face and content validity. The final draft was composed of a mix of question types to generate data for the study including multiple choice questions, Likert scale, and open-ended questions. Respondents were asked to rate potential facilitators or barriers using a 5-point Likert scale, where 1 = strongly disagree, 2 = disagree, 3 = neutral, 4 = agree, and 5 = strongly agree. A preface was included at the beginning of the survey to define CDS tools and provide links to examples used in clinical practice, and to ask the participants to tailor their responses to the care of older adults with kidney disease. The survey questions were delivered in digital format through an online platform (LimeSurvey®). The survey was conducted in English.

### Expert review

The survey was sent to four HCPs practicing in different settings and different healthcare disciplines for comprehensive content review. The experts represented various disciplines, including pharmacy, medicine, and nursing.

Participants: Physicians, pharmacists, and nurse practitioners who were currently in practice in Canada were eligible to participate.

### Ethics approval

This study was approved by Research Review Office of the CIUSSS de l’Est-de-l’Île-de-Montréal Institutional Review Board, and it was conducted according to the Declarations of Helsinki. Informed consent was obtained from all subjects before participating in the survey study.

### Participants recruitment

Potential participants were healthcare providers who were practicing in Canada. They were approached through different Canadian healthcare societies and associations, including *Société québécoise de néphrologie*, *Diabète Québec*, *Association professionnelle des pharmaciens salariés du Québec (APPSQ)*, *Association des pharmaciens des établissements de santé du Québec (APES), *CanadianSociety of Nephrology, Canadian Medical Association, Canadian Pharmacists Association and related provincial bodies, Canadian Society of Hospital Pharmacists, Canadian Nurses Association, *Association Québécoise des Infirmières et Infirmiers*, and Canadian Diabetes Association. Potential participants were contacted by email by the administrators of the associations on behalf of the researchers and were sent invitations for participation. Invitations to associations were sent at least twice, and individual reminders were sent before one week of the survey deadline. The invitations were sent in August 2021 and responses were collected in October 2021, with a target sample of a minimum of 100 participants.

### Statistical analysis

Descriptive analysis of frequencies and percentages were used to describe nominal data, while mean with standard deviation (SD) and median with interquartile range (IQR) were used to describe continuous data. Likert scale questions were analyzed by combining agree and strongly agree versus neutral, disagree, and strongly disagree. Statistical analysis of the data were conducted using Microsoft Excel® version 16.

## Results

Sixty-three HCPs completed the survey which was lower than the recruitment target of the study. Response rate could not be estimated as the authors did not have the number of professionals who received the invitations through their professional organizations.

### 1) Description of the HCPs’ clinical practice

Table 1 shows that most of the participants were physicians (60%), followed by pharmacists (22%) and nurse practitioners (17%). Most of the participants were specialized in nephrology (65%), followed by family medicine (16%) and geriatrics (11%). Almost half of the participants were practicing in teaching hospitals or academic centers (49%), while one-third were practicing in community hospitals (30%). Only 15% reported working currently in outpatient or ambulatory clinics. Almost half of the participants had practiced for less than 10 years in their area of specialization compared to one-third with experience of 10–20 years, and 22% reporting a post-residency experience of > 20 years.

**Table 1:** Participants’ demographics (*N* = 63)

Profession	n (Percentage)
Physician	38 (60.3%)
Pharmacist	14 (22.2%)
Nurse practitioners	11 (17.5%)
**Speciality**	
Nephrology	41 (65.1%)
Family medicine	10 (15.9%)
Geriatrics	7 (11.1%)
Not applicable	5 (7.9%)
Internal Medicine	3 (4.8%)
Other	2 (3.2%)
Emergency medicine	1 (1.6%)
Surgery, general	1 (1.6%)
**Practice Setting**	
Teaching hospital/academic medical centre	31 (49.2%)
Community hospital	19 (30.2%)
Outpatient/ ambulatory clinic	9 (14.3%)
Other	6 (9.5%)
Community pharmacy/drug store	4 (6.3%)
**Years of Experience**	
< 10 years	28 (44.4%)
10–20 years	21 (33.3%)
> 20 years	14 (22.2%)

### 2) The use of CDS tools in clinical practice in the care of older adults with kidney dysfunction

The preferred way to access CDS tools according to the participants was via smartphone application (73%), web-based interface (54%), integrated program in the electronic health records (EHR) (43%), then as pocket card (8%). Only 6% of the participants selected paper-based CDS tools as a preference. Two-thirds (64%) of the participants provided examples of some of the CDS tools they used in their daily practice. These tools fit into three main categories, as follows: CDS tools databases and platforms - like the Canadian Deprescribing Network, CDS tools integrated in drug information databases or medication management systems - including UpToDate®, and risk scores calculators– such as Kidney Failure Risk Equation and Framingham risk score calculators [10, 11]. More than a half of the participants (56%) reported that they documented their use of the CDS tools. Participants documented their use mainly in the patient’s file/chart, either in the clinical or progress notes or in the summary section.

### 3) Key components to include in a CDS tool

Table 2 shows that the large majority of the respondents (92%) rated the safety profile of a medication as a fairly or very important key component of the CDS tool to assist in making prescribing decisions for older adults in the care of older adults with kidney dysfunction. The effectiveness of the medication and the evidence level supporting the safety of therapy were both the second highly rated key components (89% each), while the goal of therapy was rated third (87%). In contrast, the lowest rated components were the involvement of patient’s family in taking care of the patient (38%), patient’s financial status (41%), and the cost of therapy (55%). The majority of the respondents (> 80%) agreed that the patient’s willingness to adhere to therapy, their remaining life expectancy, and their level of independence and functional capacity were key in prescribing decisions for older adults.

**Table 2:** Key Components of the CDS tool. The question was: How do you personally evaluate the importance of the following components/factors in a CDS tool that can assist you in prescribing decisions for older patients (65 years or older)?

Questions and Answers	Percentage
*Components based on the American Geriatrics Society (AGS) recommendations*	
1. Patient's remaining life expectancy	
Not at all important to slightly important	3%
Important to very important	97%
2. Patient's level of independence and functional capacity	
Not at all important to slightly important	2%
Important to very important	98%
3. Patient's quality of life	
Not at all important to slightly important	0%
Important to very important	100%
*Patient-related factors*	
4. Patient's living situation (living alone, with family, or living in a nursing home)	
Not at all important to slightly important	15%
Important to very important	85%
5. The involvement of patient's family in taking care of the patient	
Not at all important to slightly important	23%
Important to very important	77%
6. Patient's financial status	
Not at all important to slightly important	27%
Important to very important	73%
7. Patient's willingness to adhere to therapy	
Not at all important to slightly important	5%
Important to very important	95%
8. Patient's history of adherence to drug therapy	
Not at all important to slightly important	6%
Important to very important	94%
9. Patient's risk to develop a potential adverse drug reaction due to therapy	
Not at all important to slightly important	5%
Important to very important	95%
10. The goal of therapy (curative, palliative, for symptoms relief, to prevent fatal events or to prolong life)]	
Not at all important to slightly important	0%
Important to very important	100%
*Medication-related factors*	
11. Cost of therapy	
Not at all important to slightly important	13%
Important to very important	87%
12. Level of evidence of therapy's efficacy	
Not at all important to slightly important	0%
Important to very important	100%
13. The effectiveness of the medication (e.g., how much it reduces HbA1c)	
Not at all important to slightly important	4%
Important to very important	96%
14. Level of evidence of therapy's safety	
Not at all important to slightly important	0%
Important to very important	100%
15. Safety profile of the medication	
Not at all important to slightly important	0%
Important to very important	100%

### 4) Barriers and facilitators toward using CDS tools

Most participants indicated agreement or strong agreement regarding factors that enhance the utilization of CDS tools in daily practice in the care of older adults with kidney dysfunction. These factors include the validation of CDS tools (95%; median: 4, Interquartile Range (IQR): 4–5), presenting recommendations with supporting evidence (84%; median: 4, IQR: 4–5), and affiliating the tools with recognized organizations (84%; median: 4, IQR: 4–5). At least 60%of participants agreed or strongly agreed on the following facilitators to use CDS tools: Using CDS tools as part of shared decision-making with patients, the online accessibility of CDS tools, the availability of a number of recommendation options to choose from, and providing justifications with recommendations.

As presented in Table 3, respondents varied in their rating of time as a barrier to use CDS tools, as 38% strongly agreed or agreed that there is enough time to use CDS tools at the point of decision making [median: 3 (IQR: 2–4)], and 46% strongly agreed or agreed that there is enough time to read the justifications of CDS tool’s recommendations [median: 3 (IQR: 3–4)]. More participants agreed that they will only use CDS tools that require less than 5 min to use [(60%); median: 4 (IQR: 3–4)] compared to only using CDS tools that require less than 2 min [(41%); median: 3 (IQR: 2–4)]. The majority of participants felt comfortable using CDS tools in front of their colleagues [(83%); median: 4 (IQR: 4–5)] and to a lesser extent felt comfortable using the tools in front of their patients [(70%); median: 4 (IQR: 3–4)]. Using CDS tools in complex cases was not a barrier according to 73% of respondents [median: 4 (IQR: 3–5)].

**Table 3:** Barriers and facilitators toward using CDS tools. The question was: please indicate your level of agreement to the following statements.

Questions and Answers	Percentage
*Time factors*	
1. There is enough time to refer to CDS tools at the point of decision-making (i.e. during patient’s appointment, during the rounds, etc.)	
Disagree or Strongly disagree	35%
Neutral	27%
Agree or Strongly agree	38%
2. I will only use CDS tools that provide me with recommendations after asking a limited number of questions (e.g., less than 5 questions)	
Disagree or Strongly disagree	24%
Neutral	30%
Agree or Strongly agree	46%
3. I will only use CDS tools that require less than 5 min	
Disagree or Strongly disagree	19%
Neutral	21%
Agree or Strongly agree	60%
4. I will only use CDS tools that require less than 2 min	
Disagree or Strongly disagree	37%
Neutral	22%
Agree or Strongly agree	41%
*Credibility factors*	
5. I do trust CDS tools’ recommendations if they have been validated and tested	
Disagree or Strongly disagree	2%
Neutral	3%
Agree or Strongly agree	95%
6. I do trust CDS tools’ recommendations if they have been affiliated with known organizations	
Disagree or Strongly disagree	2%
Neutral	14%
Agree or Strongly agree	84%
7. I prefer to use CDS tools that are endorsed/supported by the hospital/clinic I work at	
Disagree or Strongly disagree	18%
Neutral	41%
Agree or Strongly agree	41%
*Accessibility-related factors*	
8. I prefer to use CDS tools that are available online	
Disagree or Strongly disagree	3%
Neutral	21%
Agree or Strongly agree	77%
9. I prefer using CDS tools that provide computerized recommendations	
Disagree or Strongly disagree	11%
Neutral	32%
Agree or Strongly agree	57%
10. I only use CDS tools if they are integrated into my clinical workflow	
Disagree or Strongly disagree	38%
Neutral	37%
Agree or Strongly agree	25%
*Recommendation-related factors*	
11. I am more likely to accept the CDS tools’ recommendations if they provide me with a number of options to choose from	
Disagree or Strongly disagree	3%
Neutral	33%
Agree or Strongly agree	64%
12. I am more likely to accept CDS tools’ recommendations if they were accompanied by the supporting evidence	
Disagree or Strongly disagree	2%
Neutral	14%
Agree or Strongly agree	84%
13. I am more likely to accept CDS tools’ recommendations if they were accompanied by a justification	
Disagree or Strongly disagree	3%
Neutral	14%
Agree or Strongly agree	82%
14. When I refer to CDS tools, I do have time to read the justification of the CDS tool’s recommendation	
Disagree or Strongly disagree	22%
Neutral	32%
Agree or Strongly agree	47%
*Process-related factors*	
15. I am comfortable to use CDS tools in front of my patients	
Disagree or Strongly disagree	11%
Neutral	19%
Agree or Strongly agree	70%
16. I am comfortable to use CDS tools in front of my colleagues	
Disagree or Strongly disagree	5%
Neutral	13%
Agree or Strongly agree	82%
17. I am comfortable to use CDS tools in complex cases	
Disagree or Strongly disagree	6%
Neutral	21%
Agree or Strongly agree	73%
18. I like to use CDS tools as part of shared decision-making with my patients	
Disagree or Strongly disagree	5%
Neutral	19%
Agree or Strongly agree	76%

### 5) Attitudes toward using CDS tools in practice

The vast majority (89%) of respondents reported willingness to use CDS tools in their practice [median: 4 (IQR: 4–5)]. Two-thirds [(65%); median: 4 (IQR: 3–4)] reported familiarity with one or more CDS tools that are available online, and 57% reported using CDS tools to help them with prescribing decisions [median: 4 (IQR: 2–4)]. 60% of respondents agreed that CDS tools are easy to use in daily practice [median: 4 (IQR: 3–4)].

As presented in Table 4, participants concurred (91%) that CDS tools are valuable for their ability to assist in making decisions based on EBM [median: 4 (IQR: 4–4)], in their potential to complement their clinical experience [(84%); median: 4 (IQR: 4–4)], and in improving efficiency in clinical care [(79%); median: 4 (IQR: 4–4)]. More than three-quarters of respondents perceived using CDS tools as a method to practice shared-decision making by either helping them in discussing prescribing decision with their patients or in involving their patients in decision-making process.

**Table 4:** Attitudes Toward Using CDS Tools in Practice. The question was: Please indicate your level of agreement to the following statements.

Questions and Answers	Percentage
*The use of CDS tools*	
1. Generally I find CDS tools easy to use in my daily practice	
Disagree or Strongly disagree	15%
Neutral	25%
Agree or Strongly agree	60%
2. I am willing to use CDS tools in my practice	
Disagree or Strongly disagree	2%
Neutral	10%
Agree or Strongly agree	88%
3. I am familiar with one or more CDS tools that are available online	
Disagree or Strongly disagree	21%
Neutral	14%
Agree or Strongly agree	65%
4. I use CDS tools to help me in decision-making for prescribing for my patients	
Disagree or Strongly disagree	34%
Neutral	27%
Agree or Strongly agree	39%
*The importance of CDS Tools - prescribing*	
5. CDS tools are extremely important tools to help me prescribe for all my patients	
Disagree or Strongly disagree	24%
Neutral	30%
Agree or Strongly agree	47%
6. CDS tools are extremely important tools to help me prescribe/deprescribe for older patients	
Disagree or Strongly disagree	16%
Neutral	29%
Agree or Strongly agree	55%
7. CDS tools can complement my clinical expertise	
Disagree or Strongly disagree	2%
Neutral	14%
Agree or Strongly agree	84%
8. The value of a CDS tool is its ability to assist in making challenging decisions	
Disagree or Strongly disagree	13%
Neutral	33%
Agree or Strongly agree	53%
9. The value of a CDS tool is in improving efficiency in clinical care	
Disagree or Strongly disagree	5%
Neutral	16%
Agree or Strongly agree	79%
*The importance of CDS Tools - EBM*	
10. The value of a CDS tool is its ability to assist in improving adherence to clinical practice guidelines	
Disagree or Strongly disagree	8%
Neutral	25%
Agree or Strongly agree	67%
11. CDS tools are alternatives to clinical practice guidelines	
Disagree or Strongly disagree	19%
Neutral	35%
Agree or Strongly agree	47%
12. The value of a CDS tool is its ability to assist in making decisions based on evidence-based medicine	
Disagree or Strongly disagree	0%
Neutral	10%
Agree or Strongly agree	90%
*Shared decision-making*	
13. CDS tools can help me in discussing the decision with my patients	
Disagree or Strongly disagree	0%
Neutral	19%
Agree or Strongly agree	81%
14. CDS tools can help me involve my patients in decision-making	
Disagree or Strongly disagree	6%
Neutral	24%
Agree or Strongly agree	70%

## Discussion

This survey study provided HCPs’ perspectives on what components are key to prescribing decision-making to older adults with kidney disease and what are the major facilitators and barriers toward using CDS tools in daily practice. The study presented that HCPs value the safety and effectiveness of medications when making prescribing decisions for older patients. In addition, HCPs do consider patients’ quality of life, willingness to adhere to therapy, their remaining life expectancy, and their level of independence as key components in taking prescribing decisions in this population. Factors that generally facilitated the use of Clinical Decision Support (CDS) tools included ensuring the credibility of the tool through validation and affiliation. The study presented that HCPs were willing to spare at most five minutes to use CDS tools, and the complexity of the clinical case was not a barrier to using CDS tools in daily practice. The study findings are crucial for the development of CDS tools aimed at guiding prescribing decisions for older adults with kidney disease. Factoring these key facilitators and barriers to CDS use in the care of older adults with kidney disease into the design of CDS tools may promote effective implementation.

Computerized or non-computerized CDS tools can assist with engaging patients in decision making by presenting the available options with their positive and negative effects while assisting them in relating their own personal goals with the clinical decision to be made [1]. Non-computerized tools are simple and basic decision-trees and algorithms that are included in CPGs or some digital CDS resources like UpToDate® [3, 12]. Computerized CDS tools are more comprehensive and can be integrated into the EHR and provide recommendations at the point of care [3, 12].. Subsequently, CDS tools have been used to help clinicians in making decisions for complex cases of multimorbidity and to improve decisional comfort [1, 3]. 

This was the first study to discuss the key components of CDS tools that clinicians prioritize when prescribing for older adults, particularly the safety and effectiveness of medications. This indicates that clinicians are aware that older adults are more susceptible to adverse drug events due to age-related changes in pharmacodynamics and pharmacokinetics, multimorbidity, and frailty, among others [13]. According to Clyne et al. prescribers understand the multifactorial nature of inappropriate prescribing to older adults [14]. Prosser et al. found that prescribers were more willing to start a new medication if the supporting evidence of its safety and efficacy was strong [15]. The third highly rated key component of CDS tools according to our study is the patient’s quality of life. This falls along the American Geriatrics Society’s (AGS) recommendations in its stepwise approach for prescribing to older adults with multimorbidity, to consider patients’ quality of life in prescribing decisions [16]. The AGS also recommends considering patient’s remaining life expectancy and the ability to perform activities of daily living, as the patient’s prognosis is relevant to the assessment of a medication’s risks and benefits and the burden of the prescribed medication [16]. 

The study also shows that the general attitude of HCP toward using CDS tools is positive. Two-thirds of HCP reported using CDS tools and they do value its importance in implementing evidence-based medicine into practice. Our study found that respondents are currently using CDS tools or are willing to use them. In the same line, Sayood *et a.l* found that community pharmacists were “overwhelmingly” in support of using CDS tools for antibiotic prescribing decisions and stewardship services, which was due to the perceived lack of experience or knowledge regarding all antibiotic-related inquiries [17]. In a Dutch cross-sectional survey, primary care physicians had generally positive attitudes towards using CDS systems that aid with polypharmacy treatments which were used as part of their workflow and integrated in their EHR systems [18]. However, the respondents questioned the added value of these CDS systems and reported that time constraints is a key barrier to adopting them in practice [18]. This variability in attitudes across studies could be linked to the fact that the general attitudes toward using CDS tools is affected by many factors including job satisfaction, accepting change, feeling burned-out, HCP’s education level, and the frequency of dealing with complex clinical cases of multimorbidity and polypharmacy [19, 20]. HCPs in our study perceived the use of CDS tools as valuable as they are perceived to facilitate prescribing according to evidence and improve adherence to CPGs.

The study highlighted that shared decision-making with patients is encouraged by using CDS tools according to HCPs, as it helps in discussing prescribing decisions with patients or in involving them in the decision-making process. Shared decision-making is growing in importance in health care, as demonstrated by an expansion on the published research on this topic over the last decade [21]. The advantages of adopting shared decision-making include advancing patients’ knowledge, improving clinical outcomes, and reduction of costs [22, 23]. Decision aids are one of the methods to implement shared decision-making [22, 23]. However, decision aids are insufficient in many cases in helping patients decide on their own. That is, successful shared decision-making requires active discussions between the HCP and the patient to exchange information on patient’s desires, concerns and priorities and factual information on benefits and risks of the therapeutic interventions [24]. Hence, CDS tools were perceived as efficient means to engage patients in shared decision-making processes through the initiation of such active exchange of information [24–26]. In this study, the study shows that HCPs believe that using CDS tools that assist with finding the most appropriate prescribing decision for their patients can also help in the process of shared decision-making.

Facilitators to the use of CDS tools have been reported in literature, including its ability to save time, being user-friendly and easy to understand, encouraging discussions about individual patient needs, and reporting the tool’s validity and reliability [20, 27–29]. The provision of staff training and organizational support were reported as factors that improve the acceptability of CDS tools in clinical care [20]. In contrast, CDS tools with poorly tailored design and functions, deficient integration with EHR systems, and the potential to cause ethical and trust concerns constitute barriers toward the use of CDS tools [27, 28, 30]. Our study found that the major facilitator to the use of CDS tools when prescribing for older adults is the tool’s validation status. If the tool was validated and affiliated with known and reputable organization, HCPs were more likely to use and trust the CDS tool. The respondents in our study also stated preferences to be able to read the supporting evidence for each recommendation provided by the CDS tool, as well as being able to select from different recommendation options.

Our survey has some limitations, as it included HCPs who voluntarily participated in answering the survey questions, self-selection bias towards those with greater interest in CDS tools and the topic of prescribing decisions to older adults cannot be ruled out and may impact generalizability. Additionally, it should be noted that all respondents were from Canada, and they were primarily physicians and specialized in nephrology. While the participants were approached through different Canadian healthcare societies and associations for pharmacists, nurses and physicians, most of the participants came through the Canadian Society of Nephrology. This is likely because of our patient population of interest. Other limitations include the small sample size, the hypothetical nature of our survey versus actually observing use of a CDS tool in practice and missing the opportunity to validate the survey with the help of reviews of larger group of experts. The small size of respondents and low response rate could be attributed to several factors including the length of the survey, the inadequate follow-up reminders, and the lack of small incentives. the survey may have been considered lengthy and this could have attributed to some of the neutral responses. Future research should evaluate the attitude toward the use of CDS tool in the care of other patient groups (i.e., without kidney disease) to allow for comparisons and to assess if the study responses were unique to the care of patients with kidney disease. Future research should include patients’ perception of and attitude toward the use of CDS tools in clinical practice. Further research studies should assess the impact of the use of CDS tools on prescribing to older adults in reducing inappropriate prescribing, polypharmacy, adverse health outcomes, patients’ reported outcomes among other measures.

## Conclusion

This is the first study to explore the key components of CDS tools for prescribing to older adults. Our study shows that the most important components of CDS tools are the safety and efficacy of the medication, the goal of therapy, and the patient’s quality of life. CDS tools are being used and accepted by HCPs and are valued for their assistance in engaging patients in making well-informed decisions.

### Electronic supplementary material

Below is the link to the electronic supplementary material.

Supplementary Material 1

Supplementary Material 2

## Data Availability

All data generated or analysed during this study are included in this published article and its supplementary information files.
