# Supplementary material for: Exploring key components and factors that influence the use of clinical decision- support tools for prescribing to older patients with kidney disease: the perspective of healthcare providers
Source: BMC Health Serv Res. 2024 Jan 23;24:126. doi: 10.1186/s12913-024-10568-1 (PMC10804714; doi:10.1186/s12913-024-10568-1)
Supplement: Supplementary file 1 — Supplementary Material 1 [file 12913_2024_10568_MOESM1_ESM.docx]

**Supplementary Tables**

[**Supp Table 1:** Key Components of the CDS tool. 2](#_Toc125270458)

[**Supp Table 2:** Barriers and Facilitators Toward Using CDS Tools 5](#_Toc125270459)

[**Supp Table 3:** Attitudes Toward Using CDS Tools in Practice 8](#_Toc125270460)

# **Supp Table 1:** Key Components of the CDS tool.

The question was: How do you personally evaluate the importance of the following components/factors in a CDS tool that can assist you in prescribing decisions for older patients (65 years or older)?

| **Questions and Answers** | | | **Percentage** |
| --- | --- | --- | --- |
| *Components based on the American Geriatrics Society (AGS) recommendations* | | | |
| 1. Patient's remaining life expectancy | | | |
|  | Not at all important | 0% | |
|  | Slightly important | 3% | |
|  | Important | 14% | |
|  | Fairly important | 37% | |
|  | Very important | 46% | |
|  | No opinion | 0% | |
|  |  |  | |
| 2. Patient's level of independence and functional capacity | | | |
|  | Not at all important | 0% | |
|  | Slightly important | 2% | |
|  | Important | 14% | |
|  | Fairly important | 40% | |
|  | Very important | 44% | |
|  | No opinion | 0% | |
|  |  |  | |
| 3. Patient's quality of life | |  | |
|  | Not at all important | 0% | |
|  | Slightly important | 0% | |
|  | Important | 13% | |
|  | Fairly important | 27% | |
|  | Very important | 60% | |
|  | No opinion | 0% | |
|  |  |  | |
| *Patient-related factors* | |  | |
| 4. Patient's living situation (living alone, with family, or living in a nursing home) | | | |
|  | Not at all important | 2% | |
|  | Slightly important | 13% | |
|  | Important | 24% | |
|  | Fairly important | 44% | |
|  | Very important | 17% | |
|  | No opinion | 0% | |
|  |  |  | |
| 5. The involvement of patient's family in taking care of the patient | | | |
|  | Not at all important | 2% | |
|  | Slightly important | 21% | |
|  | Important | 40% | |
|  | Fairly important | 30% | |
|  | Very important | 8% | |
|  | No opinion | 0% | |
|  |  |  | |
| 6. Patient's financial status | |  | |
|  | Not at all important | 8% | |
|  | Slightly important | 19% | |
|  | Important | 29% | |
|  | Fairly important | 21% | |
|  | Very important | 21% | |
|  | No opinion | 3% | |
|  |  |  | |
| 7. Patient's willingness to adhere to therapy | | | |
|  | Not at all important | 0% | |
|  | Slightly important | 5% | |
|  | Important | 8% | |
|  | Fairly important | 24% | |
|  | Very important | 62% | |
|  | No opinion | 2% | |
|  |  |  | |
| 8. Patient's history of adherence to drug therapy | | | |
|  | Not at all important | 0% | |
|  | Slightly important | 6% | |
|  | Important | 24% | |
|  | Fairly important | 38% | |
|  | Very important | 29% | |
|  | No opinion | 3% | |
|  |  |  | |
| 9. Patient's risk to develop a potential adverse drug reaction due to therapy | | | |
|  | Not at all important | 0% | |
|  | Slightly important | 5% | |
|  | Important | 11% | |
|  | Fairly important | 37% | |
|  | Very important | 46% | |
|  | No opinion | 2% | |
|  |  |  | |
| 10. The goal of therapy (curative, palliative, for symptoms relief, to prevent fatal events or to prolong life)] | | | |
|  | Not at all important | 0% | |
|  | Slightly important | 0% | |
|  | Important | 10% | |
|  | Fairly important | 29% | |
|  | Very important | 60% | |
|  | No opinion | 2% | |
|  |  |  | |
| *Medication-related factors* | |  | |
| 11. Cost of therapy | |  | |
|  | Not at all important | 0% | |
|  | Slightly important | 13% | |
|  | Important | 32% | |
|  | Fairly important | 22% | |
|  | Very important | 33% | |
|  | No opinion | 0% | |
|  |  |  | |
| 12. Level of evidence of therapy's efficacy | | | |
|  | Not at all important | 0% | |
|  | Slightly important | 0% | |
|  | Important | 11% | |
|  | Fairly important | 19% | |
|  | Very important | 68% | |
|  | No opinion | 2% | |
|  |  |  | |
| 13. The effectiveness of the medication (e.g., how much it reduces HbA1c) | | | |
|  | Not at all important | 2% | |
|  | Slightly important | 2% | |
|  | Important | 6% | |
|  | Fairly important | 24% | |
|  | Very important | 65% | |
|  | No opinion | 2% | |
|  |  |  | |
| 14. Level of evidence of therapy's safety | | | |
|  | Not at all important | 0% | |
|  | Slightly important | 0% | |
|  | Important | 10% | |
|  | Fairly important | 27% | |
|  | Very important | 62% | |
|  | No opinion | 2% | |
|  |  |  | |
| 15. Safety profile of the medication | |  | |
|  | Not at all important | 0% | |
|  | Slightly important | 0% | |
|  | Important | 6% | |
|  | Fairly important | 27% | |
|  | Very important | 65% | |
|  | No opinion | 2% | |

# **Supp Table 2:** Barriers and Facilitators Toward Using CDS Tools

The question was: Please indicate your level of agreement to the following statements

| **Questions and Answers** | **Percentage** |
| --- | --- |
| *Time factors* |  |
| 1. There is enough time to refer to CDS tools at the point of decision-making (i.e. during patient's appointment, during the rounds, etc) | |
| Strongly disagree | 2% |
| Disagree | 33% |
| Neutral | 27% |
| Agree | 35% |
| Strongly agree | 3% |
|  |  |
| 2. I will only use CDS tools that provide me with recommendations after asking a limited number of questions (e.g., less than 5 questions) | |
| Strongly disagree | 2% |
| Disagree | 22% |
| Neutral | 30% |
| Agree | 43% |
| Strongly agree | 3% |
|  |  |
| 3. I will only use CDS tools that require less than 5 minutes | |
| Strongly disagree | 0% |
| Disagree | 19% |
| Neutral | 21% |
| Agree | 38% |
| Strongly agree | 22% |
|  |  |
| 4. I will only use CDS tools that require less than 2 minutes | |
| Strongly disagree | 8% |
| Disagree | 29% |
| Neutral | 22% |
| Agree | 19% |
| Strongly agree | 22% |
|  |  |
| *Credibility factors* |  |
| 5. I do trust CDS tools' recommendations if they have been validated and tested | |
| Strongly disagree | 2% |
| Disagree | 0% |
| Neutral | 3% |
| Agree | 57% |
| Strongly agree | 38% |
|  |  |
| 6. I do trust CDS tools' recommendations if they have been affiliated with known organizations | |
| Strongly disagree | 0% |
| Disagree | 2% |
| Neutral | 14% |
| Agree | 56% |
| Strongly agree | 29% |
|  |  |
| 7. I prefer to use CDS tools that are endorsed/supported by the hospital/clinic I work at | |
| Strongly disagree | 2% |
| Disagree | 16% |
| Neutral | 41% |
| Agree | 25% |
| Strongly agree | 16% |
|  |  |
| *Accessibility-related factors* |  |
| 8. I prefer to use CDS tools that are available online | |
| Strongly disagree | 0% |
| Disagree | 3% |
| Neutral | 21% |
| Agree | 48% |
| Strongly agree | 29% |
|  |  |
| 9. I prefer using CDS tools that provide computerized recommendations | |
| Strongly disagree | 0% |
| Disagree | 11% |
| Neutral | 32% |
| Agree | 46% |
| Strongly agree | 11% |
|  |  |
| 10. I only use CDS tools if they are integrated into my clinical workflow | |
| Strongly disagree | 0% |
| Disagree | 38% |
| Neutral | 37% |
| Agree | 22% |
| Strongly agree | 3% |
|  |  |
| *Recommendation-related factors* | |
| 11. I am more likely to accept the CDS tools' recommendations if they provide me with a number of options to choose from | |
| Strongly disagree | 0% |
| Disagree | 3% |
| Neutral | 33% |
| Agree | 56% |
| Strongly agree | 8% |
|  |  |
| 12. I am more likely to accept CDS tools' recommendations if they were accompanied by the supporting evidence | |
| Strongly disagree | 0% |
| Disagree | 2% |
| Neutral | 14% |
| Agree | 51% |
| Strongly agree | 33% |
|  |  |
| 13. I am more likely to accept CDS tools' recommendations if they were accompanied by a justification | |
| Strongly disagree | 0% |
| Disagree | 3% |
| Neutral | 14% |
| Agree | 52% |
| Strongly agree | 30% |
|  |  |
| 14. When I refer to CDS tools, I do have time to read the justification of the CDS tool's recommendation | |
| Strongly disagree | 0% |
| Disagree | 22% |
| Neutral | 32% |
| Agree | 37% |
| Strongly agree | 10% |
|  |  |
| *Process-related factors* |  |
| 15. I am comfortable to use CDS tools in front of my patients | |
| Strongly disagree | 0% |
| Disagree | 11% |
| Neutral | 19% |
| Agree | 46% |
| Strongly agree | 24% |
|  |  |
| 16. I am comfortable to use CDS tools in front of my colleagues | |
| Strongly disagree | 0% |
| Disagree | 5% |
| Neutral | 13% |
| Agree | 51% |
| Strongly agree | 32% |
|  |  |
| 17. I am comfortable to use CDS tools in complex cases | |
| Strongly disagree | 0% |
| Disagree | 6% |
| Neutral | 21% |
| Agree | 43% |
| Strongly agree | 30% |
|  |  |
| 18. I like to use CDS tools as part of shared decision-making with my patients | |
| Strongly disagree | 0% |
| Disagree | 5% |
| Neutral | 19% |
| Agree | 52% |
| Strongly agree | 24% |
|  |  |

# **Supp Table 3:** Attitudes Toward Using CDS Tools in Practice

The question was: Please indicate your level of agreement to the following statements

| **Questions and Answers** | **Percentage** |
| --- | --- |
| *The use of CDS tools* |  |
| 1. Generally I find CDS tools easy to use in my daily practice | |
| Strongly disagree | 2% |
| Disagree | 13% |
| Neutral | 25% |
| Agree | 49% |
| Strongly agree | 11% |
|  |  |
| 2. I am willing to use CDS tools in my practice | |
| Strongly disagree | 0% |
| Disagree | 2% |
| Neutral | 10% |
| Agree | 59% |
| Strongly agree | 30% |
|  |  |
| 3. I am familiar with one or more CDS tools that are available online | |
| Strongly disagree | 5% |
| Disagree | 16% |
| Neutral | 14% |
| Agree | 49% |
| Strongly agree | 16% |
|  |  |
| 4. I use CDS tools to help me in decision-making for prescribing for my patients | |
| Strongly disagree | 10% |
| Disagree | 24% |
| Neutral | 27% |
| Agree | 32% |
| Strongly agree | 8% |
|  |  |
| *The importance of CDS Tools - prescribing* | |
| 5. CDS tools are extremely important tools to help me prescribe for all my patients | |
| Strongly disagree | 3% |
| Disagree | 21% |
| Neutral | 30% |
| Agree | 33% |
| Strongly agree | 13% |
|  |  |
| 6. CDS tools are extremely important tools to help me prescribe/deprescribe for older patients | |
| Strongly disagree | 3% |
| Disagree | 13% |
| Neutral | 29% |
| Agree | 41% |
| Strongly agree | 14% |
|  |  |
| 7. CDS tools can complement my clinical expertise | |
| Strongly disagree | 0% |
| Disagree | 2% |
| Neutral | 14% |
| Agree | 62% |
| Strongly agree | 22% |
|  |  |
| 8. The value of a CDS tool is its ability to assist in making challenging decisions | |
| Strongly disagree | 0% |
| Disagree | 13% |
| Neutral | 33% |
| Agree | 41% |
| Strongly agree | 13% |
|  |  |
| 9. The value of a CDS tool is in improving efficiency in clinical care | |
| Strongly disagree | 0% |
| Disagree | 5% |
| Neutral | 16% |
| Agree | 60% |
| Strongly agree | 19% |
|  |  |
| *The importance of CDS Tools - EBM* | |
| 10. The value of a CDS tool is its ability to assist in improving adherence to clinical practice guidelines | |
| Strongly disagree | 0% |
| Disagree | 8% |
| Neutral | 25% |
| Agree | 49% |
| Strongly agree | 17% |
|  |  |
| 11. CDS tools are alternatives to clinical practice guidelines | |
| Strongly disagree | 2% |
| Disagree | 17% |
| Neutral | 35% |
| Agree | 37% |
| Strongly agree | 10% |
|  |  |
| 12. The value of a CDS tool is its ability to assist in making decisions based on evidence-based medicine | |
| Strongly disagree | 0% |
| Disagree | 0% |
| Neutral | 10% |
| Agree | 68% |
| Strongly agree | 22% |
|  |  |
| *Shared decision-making* | |
| 13. CDS tools can help me in discussing the decision with my patients | |
| Strongly disagree | 0% |
| Disagree | 0% |
| Neutral | 19% |
| Agree | 62% |
| Strongly agree | 19% |
|  |  |
| 14. CDS tools can help me involve my patients in decision-making | |
| Strongly disagree | 0% |
| Disagree | 6% |
| Neutral | 24% |
| Agree | 51% |
| Strongly agree | 19% |
