# Supplementary material for: Exploring key components and factors that influence the use of clinical decision- support tools for prescribing to older patients with kidney disease: the perspective of healthcare providers
Source: BMC Health Serv Res. 2024 Jan 23;24:126. doi: 10.1186/s12913-024-10568-1 (PMC10804714; doi:10.1186/s12913-024-10568-1)
Supplement: Supplementary file 2 — Supplementary Material 2 [file 12913_2024_10568_MOESM2_ESM.pdf]

Exploring the Facilitators and Barriers Toward the Use of Clinical Decision- Support (CDS) Tools by Healthcare Providers

Welcome to our survey titled: Determining the perceptions of healthcare providers toward clinical decision-support tools for prescribing to older adults with diabetes

We are researchers from Université de Montreal, Maisonneuve-Rosemont Hospital Research Center, and the University of Alberta, and our goal is to develop a new clinical decision-support (CDS) tool for healthcare providers to help in prescribing, deprescribing, dose-adjusting, and monitoring certain medications for endocrinology and nephrology - related cases.

One part of the project is to engage end-users in the process of CDS tool design and validation. Therefore, we would like to invite you to be part of this process by participating in the following survey. We invite you to ask the research team any questions you may have before participating in this project.

Definition: *CDS tools are systems that provide end-users with recommendations based on knowledge and patient-specific facts to facilitate developing more informed judgments, which ultimately aim to enhance patient health.*

These are examples of CDS tools that are available:

- Proton Pump Inhibitor deprescribing algorithm- Canadian Deprescribing Network

  https://deprescribing.org/wp-content/uploads/2018/08/ppi-deprescribing-algorithm_2018_En.pdf

- Medication prescribing algorithm for the treatment of PTSD- Cardiff University School of Medicine

  https://www.phoenixaustralia.org/wp-content/uploads/2020/02/Medication-prescribing-algorithm-Appendix-To-Chapter-6-1.pdf

- Pneumococcal Vaccine Decision Tree - Zingtree®

  https://zingtree.com/host-gallery.php?gallery_id=18&persist_names=Restart&persist_node_ids=1#1

Objectives: To identify the key components of a CDS tool that are most important to healthcare professionals, as well as to understand the facilitators and barriers toward using CDS tools in daily clinical practice.

Estimated time: 10 minutes

Survey description: The survey is composed of 13 questions organized into five sections.

Deadline: Sep 30, 2021

RISKS, DISADVANTAGES AND BENEFITS ASSOCIATED WITH THE RESEARCH PROJECT: There are no risks or disadvantages associated with this study other than the time spent completing the online surveys. You will not benefit from participating in this research project, however, your responses will inform the design of a CDS tool. Ultimately, the CDS tool would have the potential to be used in daily clinical practice. You will not be financially compensated for your participation in this research project.

VOLUNTARY PARTICIPATION AND RIGHT OF WITHDRAWAL: Your participation in this research project is voluntary and anonymous. You are therefore free to refuse to participate. You may also stop completing the online questionnaire at any time.

Since this questionnaire does not provide the research team with any way to identify you, it will not be possible to ask the researchers to exclude your data after you have completed and submitted the questionnaire.

The record of your survey responses does not contain any identifying information about you. If you used an identification token to access this survey, please be assured that this token will not be stored with your responses. It is maintained in a separate database and will only be updated to indicate whether you have (or have not) completed this survey. There is no way to match identification tokens with survey responses.

CONFIDENTIALITY: All information collected in this research project will be kept confidential to the extent permitted by law. The researchers will not collect any personally identifiable information and your data will be anonymous. The research data will be kept for 7 years and may be published or discussed scientifically, but it will not be possible to identify you.

By participating in this study, you consent to the use of your data for other research projects in the field of CDS tools under the responsibility of this research team. The use of your data in other research projects will be done after these projects have been approved by a Research Ethics Board (REB) and will be subject to the same retention conditions, rules and standards as for this project.

Ethical approval: This project is approved by the Research Ethics Board of the CIUSSS de l'Est-de-l'Île-de-Montréal. (approval # **2022-2617**)

CONTACT PERSON IDENTIFICATION: If you have any questions or problems related to the research project, you can contact the researcher in charge [Jean-Philippe Lafrance, Tel: 514-252-3400 ext.7737 , jean-philippe.lafrance@umontreal.ca]. If you have any questions about your rights as a participant in this research project or if you have any complaints or comments to make, you can contact the Complaints and Service Quality Commissioner of the CIUSSS de l'Est-de-l'Île-de-Montréal at 514-252-3400, ext. 3510. The CIUSSS de l'Est-de-l'Île-de-Montréal Research Ethics Committee has approved the project and will ensure its follow-up.

CONSENT: I have read the information and consent form and understand the nature of the research project. My questions, if any, have been answered and I have been giveń adequate time to decide to participate. I understand that by completing this online questionnaire and submitting it, I am consenting to participate under the conditions stated in it.

Before you start, we would like to tell you about the next stage of our project. The Delphi Meetings.

The research team is looking for established experts in medical, pharmacy, and nursing practices to gain their views on how to construct an expert consensus on our CDS tool's recommendations, as part of the process of its validation. If you are interested in joining this expert committee to validate our novel decision-support tool, please include your email address at the end of the survey. We will follow-up with you once the survey is closed.

If you have any questions, please feel free to write us at noor.alsalemi@umontreal.ca, or jean-philippe.lafrance@umontreal.ca

There are 13 questions in this survey.

1. Description of the healthcare providers' clinical practice

1 **What is your profession?** *

Select all that apply
Please choose **all** that apply:

- [ ] Physician
- [ ] Pharmacist
- [ ] Nurse
- [ ] Other: ______

2
What are your practice specialization areas?
*

Select all that apply
Please choose **all** that apply:

- [ ] Cardiology
- [ ] Dermatology
- [ ] Emergency medicine
- [ ] Endocrinology
- [ ] Family medicine
- [ ] Geriatrics
- [ ] Internal Medicine
- [ ] Nephrology
- [ ] Neurology
- [ ] Obstetrics & Gynecology
- [ ] Orthopedics
- [ ] Psychiatry
- [ ] Surgery, general
- [ ] Not applicable
- [ ] Other: ______

3 **Where do you practice currently?** *

Select all that apply

Please choose **all** that apply:

- ☐ Teaching hospital/academic medical centre
- ☐ Community hospital
- ☐ Community pharmacy/drug store
- ☐ Outpatient/ ambulatory clinic
- ☐ Nursing home
- ☐ Other: ______

4 **How many years have you been practicing in your area of specialization (post-residency)?** *

Choose one of the following answers

Please choose **only one** of the following:

- ◯ < 10 years
- ◯ 10-20 years
- ◯ > 20 years

2. Key components of Clinical Decision Support (CDS) tools

5 **How do you personally evaluate the importance of the following components/factors in a CDS tool that can assist you in prescribing decisions for older patients (65 years or older)?** *

Please choose the appropriate response for each item:

	Not at all important	Slightly important	Important	Fairly important	Very important	No opinion
**1. Patient's remaining life expectancy**	◯	◯	◯	◯	◯	◯
**2. Patient's level of independence and functional capacity**	◯	◯	◯	◯	◯	◯
**3. Patient's quality of life**	◯	◯	◯	◯	◯	◯
**4. Patient's living situation (living alone, with family, or living in a nursing home)**	◯	◯	◯	◯	◯	◯
**5. The involvement of patient's family in taking care of the patient**	◯	◯	◯	◯	◯	◯
**6. Patient's financial status**	◯	◯	◯	◯	◯	◯
**7. Patient's willingness to adhere to therapy**	◯	◯	◯	◯	◯	◯
**8. Patient's history of adherence to drug therapy**	◯	◯	◯	◯	◯	◯
**9. Cost of therapy**	◯	◯	◯	◯	◯	◯
**10. Level of evidence of therapy's efficacy**	◯	◯	◯	◯	◯	◯
**11. The effectiveness of the medication (e.g., how much it reduces HbA1c)**	◯	◯	◯	◯	◯	◯
**12. Level of evidence of therapy's safety**	◯	◯	◯	◯	◯	◯
**13. Safety profile of the medication**	◯	◯	◯	◯	◯	◯
**14. Patient's risk to develop a potential adverse drug reaction due to therapy**	◯	◯	◯	◯	◯	◯

	Not at all important	Slightly important	Important	Fairly important	Very important	No opinion
**15. The goal of therapy (curative, palliative, for symptoms relief, to prevent fatal events or to prolong life)**	◯	◯	◯	◯	◯	◯

6
What are your preferred ways to access a CDS tool?

*

Select all that apply
Please choose **all** that apply:

- ☐ Paper-based
- ☐ Pocket card
- ☐ Phone application
- ☐ Web-based
- ☐ Integrated in the EMR
- ☐ Other: ____________

7
Do you document your use of the CDS tools in patient care activities?

*

Choose one of the following answers
Please choose **only one** of the following:

- ◯ Yes
- ◯ No
- ◯ Sometimes
- ◯ I don’t use CDS tools in my practice

8 **Where and how do you document your use of the CDS tool in your practice?**

Only answer this question if the following conditions are met:
Answer was 'Yes' *or* 'Sometimes' at question ' [KEY03]' (Do you document your use of the CDS tools in patient care activities?)

Please write your answer here:

9
Please list the top 3 CDS tools that you use in your practice, if any?

Please write your answer here:

3. Barriers and facilitators toward using CDS tools

10 **Please indicate your level of agreement to the following statements** *

Please choose the appropriate response for each item:

	Strongly disagree	Disagree	Neutral	Agree	Strongly agree
1. There is enough time to refer to CDS tools at the point of decision-making (i.e. during patient's appointment, during the rounds, etc)	◯	◯	◯	◯	◯
2. I will only use CDS tools that provide me with recommendations after asking a limited number of questions (e.g., less than 5 questions)	◯	◯	◯	◯	◯
3. I will only use CDS tools that require less than 5 minutes	◯	◯	◯	◯	◯
4. I will only use CDS tools that require less than 2 minutes	◯	◯	◯	◯	◯
5. I do trust CDS tools' recommendations if they have been validated and tested	◯	◯	◯	◯	◯
6. I do trust CDS tools' recommendations if they have been affiliated with known organizations	◯	◯	◯	◯	◯
7. I prefer to use CDS tools that are endorsed/supported by the hospital/clinic I work at	◯	◯	◯	◯	◯
8. I like to use CDS tools as part of shared decision-making with my patients	◯	◯	◯	◯	◯
9. I only use CDS tools if they are integrated into my clinical workflow	◯	◯	◯	◯	◯

	Strongly disagree	Disagree	Neutral	Agree	Strongly agree
**10. I prefer to use CDS tools that are available online**	◯	◯	◯	◯	◯
**11. I prefer using CDS tools that provide computerized recommendations**	◯	◯	◯	◯	◯
**12. I am more likely to accept the CDS tools' recommendations if they provide me with a number of options to choose from**	◯	◯	◯	◯	◯
**13. I am more likely to accept CDS tools' recommendations if they were accompanied by the supporting evidence**	◯	◯	◯	◯	◯
**14. I am more likely to accept CDS tools' recommendations if they were accompanied by a justification**	◯	◯	◯	◯	◯
**15. When I refer to CDS tools, I do have time to read the justification of the CDS tool's recommendation**	◯	◯	◯	◯	◯
**16. I am comfortable to use CDS tools in front of my patients**	◯	◯	◯	◯	◯
**17. I am comfortable to use CDS tools in front of my colleagues**	◯	◯	◯	◯	◯
**18. I am comfortable to use CDS tools in complex cases**	◯	◯	◯	◯	◯

4. General attitudes towards using CDS tools

11 **Please indicate your level of agreement to the following statements** *

Please choose the appropriate response for each item:

	Strongly disagree	Disagree	Neutral	Agree	Strongly agree
1. Generally I find CDS tools easy to use in my daily practice					
2. I am willing to use CDS tools in my practice					
3. CDS tools are extremely important tools to help me prescribe for all my patients					
4. CDS tools are extremely important tools to help me prescribe/deprescribe for older patients					
5. CDS tools are alternatives to clinical practice guidelines					
6. CDS tools can complement my clinical expertise					
7. I am familiar with one or more CDS tools that are available online					
8. I use CDS tools to help me in decision-making for prescribing for my patients					
9. I use CDS tools to help me in decision-making for diagnosing my patients					
10. The value of a CDS tool is its ability to assist in making challenging decisions					
11. The value of a CDS tool is its ability to assist in improving adherence to clinical practice guidelines					

	Strongly disagree	Disagree	Neutral	Agree	Strongly agree
**12. The value of a CDS tool is its ability to assist in making decisions based on evidence-based medicine**	◯	◯	◯	◯	◯
**13. The value of a CDS tool is in improving efficiency in clinical care**	◯	◯	◯	◯	◯
**14. CDS tools can help me in discussing the decision with my patients**	◯	◯	◯	◯	◯
**15. CDS tools can help me involve my patients in decision-making**	◯	◯	◯	◯	◯

5. Conclusion

12

Please provide any additional comments:

Please write your answer here:

13

Thank you for your participation in this survey study.

The research team is looking for established experts in medical, pharmacy, and nursing practices to gain their views on how to construct an expert consensus on a clinical decision support tool. Will you be interested in joining this expert committee to validate our novel decision-support tool? If your answer is yes, **please provide us with your email address by clicking on the link that will be shown on the following page**, after you submit the survey.

Please write your answer here:

Thank you for your participation in this survey study. We really appreciate your time and efforts in completing this survey.

Will you be interested in joining this expert committee to validate our novel decision-support tool? If your answer is yes, please provide us with your email address by clicking on the link below, and we will contact you soon.

https://forms.office.com/r/eiKEgZu4wM

04.10.2021 – 20:26
Submit your survey.
Thank you for completing this survey.
